# Increased abundance of secreted hydrolytic enzymes and secondary metabolite gene clusters define the genomes of latent plant pathogens in the *Botryosphaeriaceae*

**DOI:** 10.1186/s12864-021-07902-w

**Published:** 2021-08-04

**Authors:** Jan H. Nagel, Michael J. Wingfield, Bernard Slippers

**Affiliations:** Department of Biochemistry, Genetics and Microbiology, Forestry and Agricultural Biotechnology Institute (FABI), University of Pretoria, Pretoria, 0001 South Africa

**Keywords:** Secretome, CAZyme, Secondary metabolism, Comparative genomics, Endophyte, Plant cell wall-degrading enzymes

## Abstract

**Background:**

The *Botryosphaeriaceae* are important plant pathogens, but also have the ability to establish asymptomatic infections that persist for extended periods in a latent state. In this study, we used comparative genome analyses to shed light on the genetic basis of the interactions of these fungi with their plant hosts. For this purpose, we characterised secreted hydrolytic enzymes, secondary metabolite biosynthetic gene clusters and general trends in genomic architecture using all available *Botryosphaeriaceae* genomes, and selected Dothideomycetes genomes.

**Results:**

The *Botryosphaeriaceae* genomes were rich in carbohydrate-active enzymes (CAZymes), proteases, lipases and secondary metabolic biosynthetic gene clusters (BGCs) compared to other Dothideomycete genomes. The genomes of *Botryosphaeria*, *Macrophomina*, *Lasiodiplodia* and *Neofusicoccum,* in particular, had gene expansions of the major constituents of the secretome, notably CAZymes involved in plant cell wall degradation. The *Botryosphaeriaceae* genomes were shown to have moderate to high GC contents and most had low levels of repetitive DNA. The genomes were not compartmentalized based on gene and repeat densities, but genes of secreted enzymes were slightly more abundant in gene-sparse regions.

**Conclusion:**

The abundance of secreted hydrolytic enzymes and secondary metabolite BGCs in the genomes of *Botryosphaeria*, *Macrophomina*, *Lasiodiplodia*, and *Neofusicoccum* were similar to those in necrotrophic plant pathogens and some endophytes of woody plants. The results provide a foundation for comparative genomic analyses and hypotheses to explore the mechanisms underlying *Botryosphaeriaceae* host-plant interactions.

**Supplementary Information:**

The online version contains supplementary material available at 10.1186/s12864-021-07902-w.

## Background

Secreted hydrolytic enzymes and fungal toxins play crucial roles in enabling fungal pathogens to establish successful infections on their plant hosts. Among the secreted proteins, carbohydrate-active enzymes (CAZymes), protease and lipases are important for nutrient acquisition, as well as for the breakdown, manipulation (i.e effectors) or circumvention of host defences [1–7]. Fungal toxins are a diverse group of compounds and those most commonly found in fungal pathogens include polyketides, non-ribosomal peptides, terpenes and indole alkaloids [8]. These toxins are secondary metabolites that induce plant cell death, and for this reason, necrotrophic plant pathogens usually possess greater numbers of genes involved in secondary metabolite synthesis than biotrophic pathogens [9].

The genomes of many fungal and Oomycetes plant pathogens, especially those rich in repetitive elements, are not homogenous, but rather compartmentalized into repeat-rich, gene sparse regions and repeat poor, gene dense regions [10–13]. Genes localized to repeat-rich, gene sparse regions also have a higher rate of mutation and are often under stronger selective pressure [11, 14, 15]. This has given rise to a phenomenon referred to as ‘two-speed’ genomes, due to the stark differences in evolutionary rates between the two different types of genomic regions.

Fungi residing in the *Botryosphaeriaceae* include important plant pathogens. These fungi mostly cause diseases of woody plant species and they can impact negatively on the health of many economically and ecologically significant plant species [16, 17]. The *Botryosphaeriaceae* infect a wide range of plant hosts, most notably grapevine [18], pome and stone fruits [19], plantation forest trees such as *Eucalyptus* spp., *Pinus* spp. and *Acacia mangium* [20–22], as well as plants in their native habitats [23–26]. Many of these fungi (e.g. *B. dothidea*, *M. phaseolina, Lasiodiplodia theobromae*, *Neofusicoccum parvum*) have wide host ranges, while a few species (e.g. *Diplodia sapinea* on *Pinus* species) have narrower host ranges or are even very host-specific (e.g. *Eutiarosporella darliae, E. pseudodarliae* and *E. tritici-australis* on wheat) [27]. Many species of *Botryopshaeriaceae* are also known to occur endophytically in asymptomatic plant tissues or to have a latent pathogenic phase, where they inhabit their plant hosts in the absence of symptoms and cause disease only after the onset of stress, such as drought, frost or hail damage [16, 28].

A few recent studies have investigated secreted proteins and secondary metabolites in species of the *Botryosphaeriaceae.* Proteomic studies analyzing the secreted proteins of *Diplodia seriata* [29] and *D. corticola* [30] identified secreted proteins involved in pathogenesis. Studies of grapevine pathogens also predicted secreted CAZymes and genes involved in the production of secondary metabolites of *D. seriata* and *Neofusicoccum parvum* [31, 32], however no studies directly linking these genes to disease symptoms or plant interactions exist. Secondary metabolite biosynthetic gene clusters (BGCs) have also been shown to play a role in host range determination, e.g. in *E*. *darliae* and *E. pseudodarliae* causing white grain disorder, where the presence of a secondary metabolite biosynthetic gene cluster is likely to allow woody hosts to be infected [27]. Despite the many publicly available genomes of species of *Botryosphaeriaceae* [31–38], no comprehensive comparative studies have been undertaken using these genomes; neither have analyses been conducted to characterise secreted proteins and secondary metabolites in most of these fungi. Such studies are also hampered by the lack of publicly available genome annotations.

The manner in which plants interact with beneficial microorganisms, while at the same time restricting the negative effects of pathogens, is an important and intriguing question in plant biology [39]. One proposed model referred to as the ‘balanced antagonism model’ [40] holds that endophytism is a result of both the host plant and the fungus employing antagonistic measures against each other, in such a way that neither overwhelms the other. Disruption of this balance either results in the pathogen causing disease or in the host plant successfully killing the fungus. The model thus predicts that known endophytic species should have similar genetic repertoires to their closely related plant pathogenic relatives. This appears to be the case when considering recent comparative genomics studies conducted on endophytic fungi [41–44], although some endophytic species, e.g. *Xylonia heveae* had fewer CAZymes than expected and were more similar to mutualistic species [45]. Indeed, the above-mentioned endophytes (other than *X. heveae*) commonly had high numbers of plant cell wall degrading enzymes and secondary metabolite genes.

Despite their ubiquity as endophytes and their importance as latent pathogens, very little is known regarding how *Botryosphaeriaceae* species interact with their diverse plant hosts at a molecular level. Studies have characterized this fungus-host interaction for the most prominent of *Botryosphaeriaceae* species [31, 46–50], but such knowledge remains lacking for most species. Key questions in this regard relate to the secreted hydrolytic enzymes and secondary metabolic biosynthesis genes present in their genomes. Based on the results of previous studies on Ascomycetes that are endophytes of woody plants, we have hypothesised that these genes and gene clusters in the *Botryosphaeriaceae* will resemble those of closely related plant pathogens*.* To test this hypothesis, we compared the predicted secreted hydrolytic enzyme and secondary metabolite genes of *Botryosphaeriaceae* species with those of other Dothideomycetes. We also characterised the genome architecture of the *Botryosphaeriaceae* in terms of gene density, repeat content and prevalence of repeat-induced point mutations (RIP), and considered how these associate with secreted hydrolytic enzymes and secondary metabolite BGCs.

## Results

### Genome sequencing, assembly and annotation

Nine genomes of *Neofusicoccum* and three genomes of *Lasiodiplodia* species were sequenced using Illumina sequencing (Table 1). These included two isolates each of *N. cordaticola*, *N. kwambonambiense, N. parvum* and *N. ribis* were sequenced. A single isolate was sequenced for *L. gonubiensis, L. pseudotheobromae, L. theobromae* and *N. umdonicola*.

**Table 1:** List of genome sequences used in this study^a^

Species	Reference collection/Isolate number	Assembly Size (Mbp)	Number of gene models	Genome accession number	Reference
Dothideomycetes					
Botryosphaeriales					
*Botryosphaeria kuwatsukai*	LW030101	47.39	11,278	MDSR01000000	[35, 51]
*Botryosphaeria dothidea*	CMW8000	43.50	11,368	http://genome.jgi.doe.gov/Botdo1_1/Botdo1_1.home.html	[36]
*Diplodia corticola*	CBS112549	34.99	9376	MNUE01000001	[30]
*Diplodia sapinea*	CBS117911	36.05	9589	AXCF00000000	[52]
	CBS138184	35.24	9386	JHUM00000000	[52]
*Diplodia scrobiculata*	CBS139796	34.93	9204	LAEG00000000	[53]
*Diplodia seriata*	UCDDS831	37.12	9759	MSZU00000000	[32]
	F98.1	37.27	9832	LAQI00000000	[37]
*Eutiarosporella darliae*	2G6	27.27	7904	GFXH01000000	[27]
*Eutiarosporella pseudodarliae*	V4B6	26.74	7846	GFXI01000000	[27]
*Eutiarosporella tritici-australis*	153	26.59	7783	GFXG01000000	[27]
***Lasiodiplodia gonubiensis***	**CBS115812**	41.14	10,649	**RHKH00000000**	Present study
***Lasiodiplodia pseudotheobromae***	**CBS116459**	43.01	10,964	**RHKG00000000**	Present study
***Lasiodiplodia theobromae***	**CBS164.96**	42.97	10,961	**RHKF00000000**	Present study
	CSS01	43.28	11,017	RHKB00000000	[49]
*Macrophomina phaseolina*	MS6	48.88	10,799	AHHD00000000	[34]
***Neofusicoccum cordaticola***	**CBS123634**	45.71	12,822	**RHKC00000000**	Present study
	**CBS123638**	43.56	12,630	**RHKD00000000**	Present study
***Neofusicoccum kwambonambiense***	**CBS123639**	44.17	12,839	**RHKE00000000**	Present study
	**CBS123642**	44.21	12,904	**RKSS00000000**	Present study
***Neofusicoccum parvum***	**CMW9080**	41.41	12,870	**RHJX00000000**	Present study
	**CBS123649**	42.16	12,453	**RHJY00000000**	Present study
	UCRNP2	42.52	12,691	AORE00000000	[33]
***Neofusicoccum ribis***	**CBS115475**	43.18	12,708	**RHJZ00000000**	Present study
	**CBS121.26**	43.12	12,733	**RHKA00000000**	Present study
***Neofusicoccum umdonicola***	**CBS123644**	42.29	12,816	**RHKB00000000**	Present study
Capnodiales					
*Acidomyces richmondensis*	meta	26.82	10,338	JOOL00000000	[54]
*Baudoinia panamericana*	UAMH 10762	21.88	10,508	AEIF00000000	[4]
*Cercospora berteroae*	CBS538.71	33.89	11,903	PNEN00000000	[55]
*Cercospora beticola*	09–40	37.06	12,463	LKMD00000000	[55]
*Cercospora zeina*	CMW25467	40.76	10,193	MVDW00000000	[56]
*Dothistroma septosporum*	NZE10	30.21	12,415	AIEN00000000	[4]
*Pseudocercospora eumusae*	CBS 114824	47.12	12,632	LFZN01000000	[57]
*Pseudocercospora fijiensis*	CIRAD86	29.98	13,066	AIHZ00000000	[4]
*Pseudocercospora musae*	CBS 116634	60.44	13,129	LFZO00000000	[57]
*Ramularia collo-cygni*	URUG2	32.25	11,612	FJUY00000000	[58]
*Sphaerulina musiva*	SO2202	29.35	10,233	AEFD00000000	[4]
*Zymoseptoria tritici*	IPO323	39.69	10,963	ACPE00000000	[59]
Dothideales					
*Aureobasidium namibiae*	CBS 147.97	25.43	10,259	AYEM00000000	[60]
*Aureobasidium subglaciale*	EXF-2481	25.80	10,792	AYYB00000000	[60]
Hysteriales					
*Hysterium pulicare*	CBS 123377	38.43	12,352	AJFK00000000	[4]
*Rhytidhysteron rufulum*	CBS 306.38	40.18	12,117	AJFL00000000	[4]
Myriangiales					
*Elsinoe australis*	NL1	23.34	9223	NHZQ00000000	[7]
Mytilinidiales					
*Lepidopterella palustris*	CBS 459.81	45.67	13,861	LKAR00000000	[61]
Pleosporales					
*Alternaria alternata*	SRC1lrK2f	32.99	13,466	LXPP00000000	[62]
*Ascochyta rabiei*	ArDII	34.66	10,596	JYNV00000000	[63]
*Bipolaris maydis*	ATCC 48331	32.93	12,705	AIHU00000000	[4]
*Bipolaris oryzae*	ATCC 44560	31.36	12,002	AMCO00000000	[64]
*Bipolaris sorokiniana*	ATCC 44560	34.41	12,214	AEIN00000000	[64]
*Bipolaris victoriae*	FI3	32.83	12,882	AMCY00000000	[64]
*Bipolaris zeicola*	26-R^− 13^	31.27	12,853	AMCN00000000	[64]
*Clohesyomyces aquaticus*	CBS 115471	49.68	15,811	MCFA00000000	[65]
*Corynespora cassiicola*	CCP	44.85	17,158	NSJI00000000	[66]
*Epicoccum nigrum*	ICMP 19927	34.74	12,025	NCTX00000000	[67]
*Exserohilum turcicum*	Et28A	43.01	11,698	AIHT00000000	[4]
*Leptosphaeria maculans*	JN3	45.12	12,469	FP929064:FP929139	[12]
*Paraphaeosphaeria sporulosa*	AP3s5-JAC2a	38.46	14,734	LXPO00000000	[62]
*Parastagonospora nodorum*	SN15	37.21	15,994	AAGI00000000	[68]
*Periconia macrospinosa*	DSE2036	54.99	18,735	PCYO00000000	[69]
*Pyrenophora tritici-repentis*	Pt-1C-BFP	38.00	12,169	AAXI00000000	[70]
*Stemphylium lycopersici*	CIDEFI 216	35.17	8997	LGLR00000000	[71]
Venturiales					
*Verruconis gallopava*	CBS 43764	31.78	11,357	JYBX00000000	[72]
*incertae sedis*					
*Cenococcum geophilum*	1.58	177.56	14,709	LKKR00000000	[61]
*Coniosporium apollinis*	CBS 100218	28.65	9308	AJKL00000000	[72]
*Glonium stellatum*	CBS 207.34	40.52	14,277	LKAO00000000	[61]
Eurotiomycetes					
Eurotiales					
*Aspergillus nidulans*	FGSC A4	30.28	9556	AACD00000000	[73]

^a^Entries in boldface represent genomes that were sequenced as part of the present study

De novo genome assembly resulted in genome lengths of approximately 43 MB for both *Lasiodiplodia* spp. and *Neofusicoccum* spp. (Table 2). The number of scaffolds/contigs was variable between the sequenced genomes, but the three *Lasiodiplodia* genomes had a lower number of scaffolds (376–424) than the *Neofusicoccum* genomes (1343–5188). The *N. parvum* CMW9080 genome that was sequenced on the Miseq platform had a higher degree of fragmentation, as seen from the high total number of scaffolds (5188) and a large number of short contigs (N50: 897, L50:13.55 kb) and scaffolds (N50:830, L50: 14.81 kb). The percentage of repetitive elements of each genome was significantly greater in the *Neofusicoccum* genomes (6.84%) than in the *Lasiodiplodia* genomes (3.33%) (*p* = 0.004545, Wilcoxon rank sum test).

**Table 2:** Genome statistics of new draft *Botryosphaeriaceae* genomes

	# scaffolds	# contigs	Scaffold length (Mb)	Contig length (Mb)	Genome scaffold N50/L50 (#/kb)	Genome contig N50/L50 (#/kb)	Maximum scaffold length (Mb)	Maximum contig length (kb)	% main genome in scaffolds > 50 KB
*Lasiodiplodia gonubiensis* CBS115812	376	1578	41.14	40.97	50/234.83	267/45.4	1.06	226.76	0.92
*Lasiodiplodia pseudotheobromae* CBS116459	403	1285	43.01	42.89	48/236.23	218/61.88	1.03	416.59	0.91
*Lasiodiplodia theobromae* CBS164.96	424	1093	42.97	42.88	48/223.84	163/81.56	1.69	591.21	0.91
*Neofusicoccum cordaticola* CBS123634	1912	8698	45.71	45.10	276/47.38	1218/10.76	680.02	143.71	0.48
*Neofusicoccum cordaticola* CBS123638	2393	3252	43.56	43.44	254/51.16	386/33.04	274.15	152.21	0.51
*Neofusicoccum kwambonambiense* CBS123639	1560	2839	44.17	43.99	219/59.44	363/34.21	344.53	186.24	0.58
*Neofusicoccum kwambonambiense* CBS123642	1717	2871	44.21	44.06	210/61.88	352/34.6	387.07	262.94	0.58
*Neofusicoccum parvum* CBS123649	2185	6686	42.16	41.76	312/39.13	995/12.5	439.22	92.09	0.40
*Neofusicoccum parvum* CMW9080	5188	5739	41.41	41.39	830/14.81	897/13.55	99.22	80.57	0.04
*Neofusicoccum ribis* CBS115475	1994	3261	43.18	43.03	301/42.08	474/26.80	231.82	186.29	0.43
*Neofusicoccum ribis* CBS121.26	2417	3145	43.12	43.03	245/51.65	371/33.92	280.49	167.59	0.51
*Neofusicoccum umdonicola* CBS123644	1343	2424	42.29	42.15	165/73.88	345/36.48	422.57	210.10	0.67

Twenty-six *Botryosphaeriaceae* genomes were annotated by predicting protein-coding genes with MAKER using BRAKER trained profiles. BUSCO analysis using the Ascomycota ortholog library (Table 3) indicated that all *Botryosphaeriaceae* genomes had a high degree of completeness (average of 98.%, minimum of 95.1%). The genome annotations that were generated also had a high BUSCO completeness score (average of 97.8.%, minimum of 94.3%). When comparing theses BUSCO results to those of species with existing genome annotations on NCBI/JGI, it was clear that in five out of the six cases the genome annotations from the present study had a higher BUSCO completeness score than the existing genome annotations.

**Table 3:** Genome and genome annotation completeness assesment^a^

	Genome BUSCO %					Current annotation BUSCO %					Prior annotation BUSCO %				
Genome	C	S	D	F	M	C	S	D	F	M	C	S	D	F	M
*Botryosphaeria kuwatsukai* LW030101	99.2	98.9	0.3	0.1	0.7	98.4	98.1	0.3	0.6	1.0					
*Botryosphaeria dothidea* CMW8000	99.0	98.6	0.4	0.3	0.7	98.5	98.1	0.4	0.5	1.0	98.1	97.7	0.4	0.3	1.6
*Diplodia corticola* CBS112549	98.9	98.7	0.2	0.1	1.0	98.7	98.6	0.1	0.5	0.8	99.4	99.2	0.2	0.3	0.3
*Diplodia sapinea* CBS117911	98.0	97.8	0.2	0.5	1.5	97.6	97.5	0.1	0.9	1.5					
*Diplodia sapinea* CBS138184	96.0	95.8	0.2	0.8	3.2	95.6	95.4	0.2	1.2	3.2					
*Diplodia scrobiculata* CBS139796	95.4	95.2	0.2	2.3	2.3	95.0	94.8	0.2	1.8	3.2					
*Diplodia seriata* UCDDS831	99.1	98.9	0.2	0.2	0.7	98.8	98.7	0.1	0.5	0.7	87	86.9	0.1	5.9	7.1
*Diplodia seriata* F98.1	99.3	98.9	0.4	0.1	0.6	98.9	98.5	0.4	0.5	0.6	90.1	89.9	0.2	2.6	7.3
*Eutiarosporella darliae* 2G6	98.1	97.8	0.3	0.6	1.3	98.5	97.8	0.7	0.5	1.0					
*Eutiarosporella pseudodarliae* V4B6	98.2	98.0	0.2	0.6	1.2	98.1	97.8	0.3	0.7	1.2					
*Eutiarosporella tritici-*australis 153	98.3	98.0	0.3	0.4	1.3	98.6	98.4	0.2	0.4	1.0					
*Lasiodiplodia gonubiensis* CBS115812	97.2	97.1	0.1	0.2	2.6	98.3	98.2	0.1	0.8	0.9					
*Lasiodiplodia pseudotheobromae* CBS116459	99.1	98.9	0.2	0.2	0.7	98.3	98.0	0.3	0.8	0.9					
*Lasiodiplodia theobromae* CBS164.96	97.2	97.0	0.2	0.2	2.6	98.6	98.2	0.4	0.5	0.9					
*Lasiodiplodia theobromae* CSS01	99.1	98.7	0.4	0.0	0.9	99.1	98.7	0.4	0.4	0.5					
*Macrophomina phaseolina* MS6	98.9	98.7	0.2	0.0	1.1	98.1	97.9	0.2	0.8	1.1	91.2	91	0.2	4.5	4.3
*Neofusicoccum cordaticola* CBS123634	97.0	94.9	2.1	0.9	2.1	96.1	94.2	1.9	1.6	2.3					
*Neofusicoccum cordaticola* CBS123638	98.5	98.0	0.5	0.5	1.0	98.3	97.9	0.4	0.6	1.1					
*Neofusicoccum kwambonambiense* CBS123639	99.0	98.6	0.4	0.3	0.7	98.6	98.2	0.4	0.7	0.7					
*Neofusicoccum kwambonambiense* CBS123642	98.9	98.4	0.5	0.4	0.7	98.8	98.3	0.5	0.5	0.7					
*Neofusicoccum parvum* CMW9080	95.4	95.0	0.4	2.0	2.6	94.3	93.9	0.4	2.0	3.7					
*Neofusicoccum parvum* CBS123649	95.1	94.5	0.6	0.9	4.0	96.4	95.9	0.5	1.3	2.3					
*Neofusicoccum parvum* UCRNP2	98.1	97.9	0.2	0.5	1.4	97.8	97.6	0.2	0.9	1.3	84.9	84.8	0.1	6.6	8.5
*Neofusicoccum ribis* CBS115475	98.3	97.9	0.4	0.9	0.8	97.6	97.2	0.4	1.3	1.1					
*Neofusicoccum ribis* CBS121.26	98.7	98.3	0.4	0.6	0.7	98.2	97.7	0.5	1.2	0.6					
*Neofusicoccum umdonicola* CBS123644	98.7	98.2	0.5	0.5	0.8	98.1	97.5	0.6	1.1	0.8					

^a^BUSCO percentages for Complete (C), single (S), duplicated (D), fragmented (F) and missing (M) genes. Assesments were done using the Ascomycota ortholog library

### Phylogenomic analyses

**Fig. 1:**
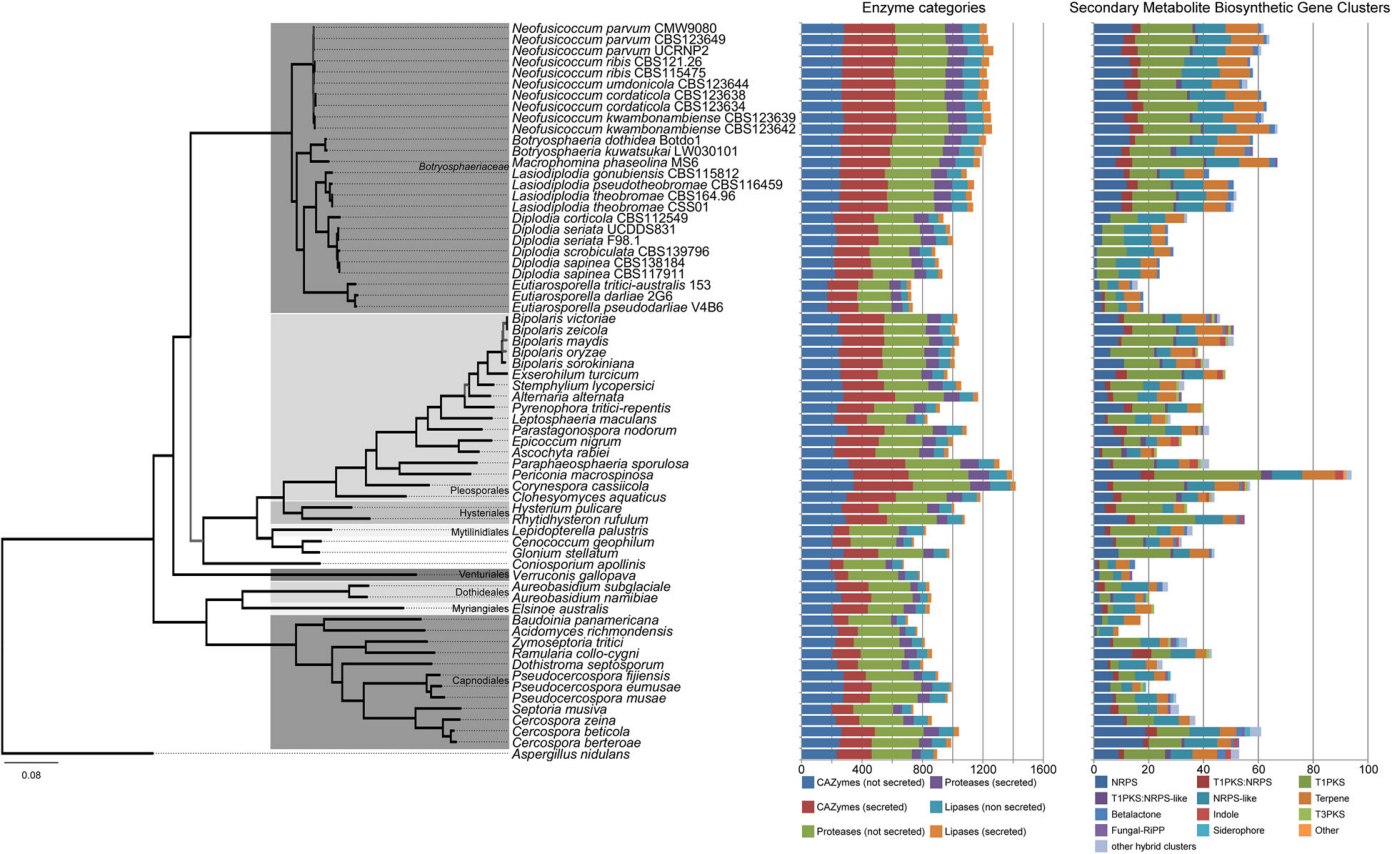
Phylogenomic tree and functional annotation summary of 26 *Botryosphaeriaceae,* 39 Dothideomycetes and one outgroup (*Aspergillus nidulans*). The supermatrix maximum likelihood phylogeny was determined using the sequence data of 207 single-copy core orthologous genes. Branches with 100% bootstrap support are indicated in black, those with less than 100% support are indicated in grey. This phylogeny illustrates the how the *Botryosphaeriaceae* taxa are related to one another, as well as how the *Botryosphaeriaceae* relates to the other Dothideomycetes. The number of genes (secreted and non-secreted) annotated with CAZyme, proteases or lipase activity, as well as the number of gene cluster types involved in secondary metabolite biosynthesis are indicated using bar graphs. These bar graphs depict that the number of these functional annotations are generally conserved within each *Botryosphaeriaceae* genus but that these values vary widely across this family. The *Botryosphaeriaceae* contains some of the largest (*Neofusicoccum*) as well as some of the smallest (*Eutiarosporella*) amounts of these functional annotations among the Dothideomycetes. Further statistical analyses of these values are provided in Additional file 2

We identified 207 core orthologous genes from the collection of 26 *Botryosphaeriaceae*, 39 other Dothideomycetes and the outgroup (*Aspergillus nidulans*) genomes. Only orthologous genes that were represented by a single gene per species were retained. The results of the phylogenomic analyses corresponded well to previous phylogenies for the Dothideomycetes [74–76]. The phylogeny indicated the early divergence of the Dothideomycetidae (Dothideales, Capnodiales and Myrangiales) from the lineage containing the Pleosporomycetidae (Pleosporales, Hysteriales and Mytilinidiales) and other Dothideomycetes without current subclass designation (Fig. 1). This phylogeny further supported the early divergence of the Botryosphaeriales from the ancestral Dothideomycetes lineage after the divergence of the Dothideomycetidae and Venturiales. The phylogenetic relationships between the *Botryosphaeriacaeae* were well defined and the phylogenetic placement of species and genera corresponded with that found in previous studies [77, 78].

### Functional annotation

In the *Botryosphaeriaceae,* both genome size and the total gene number were strongly correlated with the number of secreted proteins, CAZymes, proteases, lipases and secondary metabolite gene clusters (Additional files 1 and 2). Within the Dothideomycetes, however, the numbers of secreted proteins, CAZymes, proteases, lipases, and secondary metabolite BGCs present within a genome were correlated with one another (i.e. species that contained large numbers of secreted proteins also contained large numbers of CAZymes, proteases, lipases and secondary metabolite BGCs), but only weakly correlated with genome size and the total number of genes (Additional file 2).

**Fig. 2:**
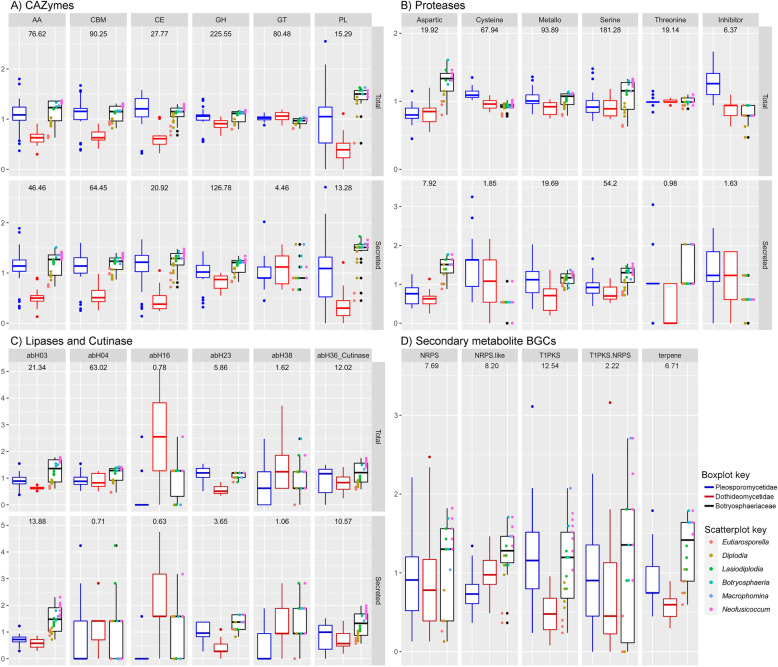
Box-and-whisker plots of the number of prominent CAZyme, protease and lipase classes (total and secreted) and secondary metabolite BGC types present within the genomes of the considered *Botryosphaeriaceae* and other Dothideomycetes taxa. Represented data is scaled using the mean of each class/type and is indicated by the number appearing at the top of each facet. Taxa are placed into three categories: Dothideomycetidae, Pleosporomycetidae (plus related taxa without subclass designation) and *Botryosphaeriaceae*. The upper and lower bounds of the box represent the 1st and 3rd quartiles (respectively) and the bar inside represent the median. The error lines (whiskers) represents 1.5 times the interquartile range (IQR) and outliers are indicated as dots. Additionally, the genera of the *Botryosphaeriaceae* are indicated by means of a scatterplot overlain on the *Botryosphaeriaceae* box-and-whisker plot. These graphs visually compares the variance of each functional annotation class between the *Botryosphaeriaceae* and other Dothideomycetes subclasses. It also depicts that, among the *Botryosphaeriaceae*, the *Eutiarosporella* and *Diplodia* have smaller amounts of most functional annotation classes than the other genera

Among the *Botryosphaeriaceae,* the *Eutiarosporella* spp. had the lowest number of each of functional annotation category, followed in increasing order by *Diplodia* spp. and *Botryosphaeria*, *Lasiodiplodia*, *Macrophomina,* and *Neofusicoccum* species (Fig. 1, Additional file 1). This observation was also evident when considering the different classes of CAZymes/proteases/lipases and the different types of secondary metabolite BGCs (Fig. 2). Furthermore, for many functional annotation categories the *Botryosphaeriaceae* were more similar to the Pleosporomycetidae than the Dothideomycetidae.

The genomes of the *Botryosphaeriaceae* genera differed significantly (*p*-value < 0.05) for many of the annotation categories (Additional file 2). All genera were significantly different (*Neofusicoccum > Botryosphaeria-*clade > *Lasiodiplodia* > *Diplodia* > *Eutiarosporella*) for the number of secreteted genes, number of total CAZymes and secreted CAZymes. This trend also existed for the other annotation categories with a few exceptions: Among the proteases and lipases, the *Neofusicoccum* and *Botryosphaeria*-clade were not significantly different. When considering the secreted proteases, species in the *Botryosphaeria*-clade were not significantly different to those of *Lasiodiplodia*, *Eutiarosporella* or *Diplodia*. The secreted lipases were not significantly different between *Eutiarosporella* and *Diplodia*. The secondary metabolite BGCs were not significantly different between the *Neofusicoccum* and species in the *Botryosphaeria*-clade.

Significant differences also existed when comparing the functional annotation categories of the *Botryosphaeriaceae* to the rest of the Dothideomycetes (Additional file 2). The *Botryosphaeriaceae* had significantly greater numbers of secreted genes, total CAZymes and total proteases than the Dothideomycetidae. Furthermore, the *Botryosphaeriaceae* had significantly greater numbers of secreted CAZymes, secreted proteases, both total and secreted lipases and secondary metabolite BGCs than both Dothideomycetidae and Pleosporomycetidae.

The *Botryosphaeriaceae,* had significantly more CAZymes of the auxiliary activities (AA), carbohydrate-binding modules (CBM), carbohydrate esterases (CE) and glycoside hydrolases (GH) classes than Dothideomycetidae (Fig. 2, Additional file 2). Additionally, the *Botryosphaeriaceae* had significantly more polysaccharide lyase (PL) genes than both Dothideomycetidae and Pleosporomycetidae. Conversely, significantly fewer CEs were present in the *Botryosphaeriaceae* than in the Pleosporomycetidae, as well as fewer glycosyltransferases (GT) than the other two Dothideomycetes sub-classes.

When considering the secreted CAZyme classes, the *Botryosphaeriaceae* had significantly more CAZymes of the AA, CBM and CE classes than Dothideomycetidae and significantly more GH and PL classes than both Dothideomycetidae and Pleosporomycetidae (Fig. 2, Additional file 2). The most abundant secreted CAZyme families in the *Botryosphaeriaceae* were CBM1, AA3, GH3, GH43, GH5, AA9, CBM18, AA1, GH28 and CBM13 (Additional file 1).

The *Botryosphaeriaceae* had above-average numbers of aspartic- (A), metallo- (M) and serine- (S) proteases, especially in the species of *Botryosphaeria*, *Lasiodiplodia*, *Macrophomina,* and *Neofusicoccum* (Fig. 2)*.* For both the total and secreted number of predicted aspartic and serine proteases, the *Botrosphaeriaceae* had significantly greater levels than the Dothideomycetidae and Pleosporomycetidae (Fig. 2, Additional file 2). The total and secreted metallo-proteases of the *Botryosphaeriaceae* were significantly higher than those of the Dothideomycetidae. These three protease classes were also the dominant proteases in the secretome. Furthermore, the *Botryosphaeriaceae* had significantly fewer secreted cysteine (C) proteases and protease inhibitors (I) than the other Dothideomycetes. Notably, the *Botryosphaeriaceae* possessed a single secreted protease inhibitor family, namely I51.001 (serine carboxypeptidase Y inhibitor), whereas many other Dothideomycetes secreted protease inhibitors were of this family, as well as I09.002 (peptidase A inhibitor 1) or I09.003 (peptidase B inhibitor). *Diplodia sapinea* and *D. scrobiculata* had no secreted protease inhibitors. The most abundant secreted protease families among the *Botryosphaeriaceae* were S09, A01, S10, S08, M28, S53, S33, M43, M35 and S12 (Additional file 1).

In the *Botryosphaeriaceae* and the Dothideomycetes, the most abundant lipases/lipase-like families were abH04 (*Moraxella* lipase 2 like), abH03 (*Candida rugosa* lipase-like), abH36, (cutinase) and abH23 (Filamentous fungi lipases) (Fig. 2). The abH03, abH36 and abH23 lipase families were the main constituents of the predicted secretomes among the Dothideomycetes and the *Botryosphaeriaceae* had significantly more of these secreted enzymes than the Dothideomycetidae and Pleosporomycetidae (Additional file 2).

The *Botryosphaeriaceae* genomes were rich in gene clusters involved in the synthesis of secondary metabolites (Fig. 2). Type 1 polyketide synthases (t1PKS) were the most abundant type of gene cluster, followed by non-ribosomal peptide synthetases (NRPS) and NRPS-like, terpene synthases (TS) and t1PKS-NRPS hybrid clusters. The *Botryosphaeriaceae* had significantly more t1PKS clusters than the Dothideomycetidae and more NRPS-like, TS and betalactone clusters than both Dothideomycetidae and Pleosporomycetidae (Additional file 2). Certain Dothideomycetes genomes, predominantly those of the Pleosporomycetidae also contained indole and type 3 PKS BGCs, however, these were not present in any of the *Botryosphaeriaceae* genomes.

The most abundant secreted CAZyme, protease and lipase families of the *Botryosphaeriaceae* were also those that had the greatest difference from the rest of the Dothideomycetes (Table 4, Additional file 2). The twenty most abundant secreted hydrolytic enzyme families of the *Botryosphaeriaceae* were all significantly greater that both the Dothideomycetidae and Pleosporomycetidae, with the exception of the secreted CBM1 and AA9 CAZymes and the M28 metalloprotease families of the *Botryosphaeriaceae* that were not significantly greater than those of the Pleosporomycetidae. Furthermore, these twenty most abundant secreted families of the *Botryosphaeriaceae* were also those that had, on average, the highest deviation from the Dothideomycetes average. The number of genes in these gene families were not increased among the non-secreted proteins. Consequently, the ratio of secreted to total proteins for these gene families was higher among the *Botryosphaeriaceae* than in the the Dothideomycetidae and Pleosporomycetidae.

**Table 4:** Comparison between the secreted and non-secreted amounts of the twenty most abundant secreted enzyme families in the *Botryosphaeriaceae*

Enzyme family	CBM1	S09	AA3	abH03	abH36	GH3	GH43	GH5	A01	AA9	CBM18	S10	AA1	GH28	S08	CBM13	M28	PL1	CE5	PL3
Amount of secreted proteins																				
*Botryosphaeriaceae* average	42.85	28.96	22.62	20.50	13.62	12.92	12.54	11.38	11.38	10.92	10.73	10.50	9.81	8.73	8.23	7.69	7.38	7.35	7.23	5.96
Deviation from the Dothideomycetes average^a^	13.77	12.19	8.85	11.04	5.08	5.15	5.97	3.08	5.77	0.33	4.01	2.09	3.86	4.09	2.10	3.05	0.90	3.12	2.85	3.45
Significantly greater than^b^	D	D, P	D, P	D, P	D, P	D, P	D, P	D, P	D, P	D	D, P	D, P	D, P	D, P	D, P	D, P	D	D, P	D, P	D, P
Significantly less than^b^										P										
Amount of non-secreted proteins																				
*Botryosphaeriaceae* average	4.73	52.00	12.50	6.85	0.62	10.58	2.12	4.65	10.92	0.85	1.73	3.42	9.54	0.77	3.42	1.15	3.96	0.19	0.27	1.46
Deviation from the Dothideomycetes average^a^	−0.60	6.49	0.47	−1.03	−1.38	3.47	−2.68	−1.17	1.87	−1.08	−2.91	0.63	4.26	−0.41	−0.91	0.08	−1.78	−0.17	−0.99	1.00
Significantly greater than^b^		D, P	D	D		D, P			P			P	D, P							D, P
Significantly less than^b^				P	D, P		P	D, P		P	P			D	P		D, P	P	D, P	
Ratio of secreted to total predicted amounts																				
Dothideomycetidae	0.79	0.23	0.48	0.61	0.75	0.59	0.57	0.52	0.34	0.88	0.54	0.71	0.48	0.63	0.61	0.73	0.43	0.94	0.76	0.30
Pleosporomycetidae	0.81	0.29	0.53	0.53	0.79	0.46	0.53	0.59	0.42	0.85	0.61	0.76	0.54	0.88	0.56	0.83	0.55	0.91	0.76	0.94
*Botryosphaeriaceae*	0.90	0.36	0.63	0.76	0.95	0.56	0.85	0.71	0.51	0.93	0.87	0.76	0.51	0.93	0.72	0.88	0.65	0.97	0.95	0.81
*Eutiarosporella*	0.94	0.42	0.61	0.97	0.95	0.59	0.75	0.68	0.47	0.97	0.89	0.86	0.58	1.00	0.72	0.95	0.67	1.00	0.94	0.89
*Diplodia*	0.91	0.32	0.56	0.74	0.89	0.59	0.81	0.67	0.49	0.88	0.89	0.85	0.54	0.96	0.83	0.87	0.65	0.97	0.87	0.81
*Lasiodiplodia*	0.88	0.36	0.61	0.73	1.00	0.59	0.88	0.70	0.49	0.81	0.89	0.76	0.49	0.90	0.68	0.83	0.59	0.98	1.00	0.75
*Botryosphaeria-clade*	0.88	0.32	0.67	0.70	0.94	0.61	0.86	0.72	0.59	0.92	0.92	0.71	0.52	0.93	0.79	0.80	0.61	0.96	1.00	0.71
*Neofusicoccum*	0.90	0.37	0.68	0.75	0.97	0.49	0.88	0.74	0.52	0.99	0.82	0.70	0.48	0.89	0.66	0.90	0.68	0.98	0.98	0.84

^a^ Deviation from the Dothideomycetes average excluding the *Botryosphaeriaceae*

^b^ Dothideomycete subclass designation: *D* Dothideomycetidae, *P* Pleosporomycetidae

### Gene family evolution

These results of the CAFE analyses (Additional file 3) indicated that expansions and contractions of CAZyme gene families occurred at roughly similar levels. This was after the divergence of the Botryosphaeriales ancestor from the Pleosporomycetidae until the formation of the *Botryosphaeriaceae* crown group (61 MYA) [79]. During this time, protease gene families experienced more contractions than expansions, lipase gene families had slightly more expansions than contractions and secondary metabolite BGCs experienced a large amount of gene family contractions. Several CAZyme gene families (AA1, AA3, AA7, AA8, AA9, CBM1, CBM18, CE4, CE5, GH3, GH10, GH28, GH43, GH78, GT1, GT2, GT25, PL1 and PL3) experienced rapid expansion (i.e. greater than expected under the birth/death model of gene family evolution) prior to the divergence of the *Botryosphaeriaceae* crown group.

After the divergence of the *Botryosphaeriaceae,* the genera *Botryosphaeria, Lasiodiplodia, Macrophomina* and *Neofusicoccum* experienced more gene family expansions than contractions, whereas the opposite was observed for the *Diplodia* and *Eutiariosporella*. Among the *Neofusicoccum* spp., the AA3, AA7, GH3 and GT2 gene families were rapidly expanding. Similarly, among the *Lasiodiplodia* spp., the AA7 and GH106 gene families were rapidly expanding. Conversely, among the *Diplodia* spp. the AA7 gene family was rapidly contracting, as were the AA1, AA3, AA7, GH28, PL1 and PL3 gene families among the *Eutiarosporella* spp.

### Principal component analysis and hierarchical clustering

**Fig. 3:**
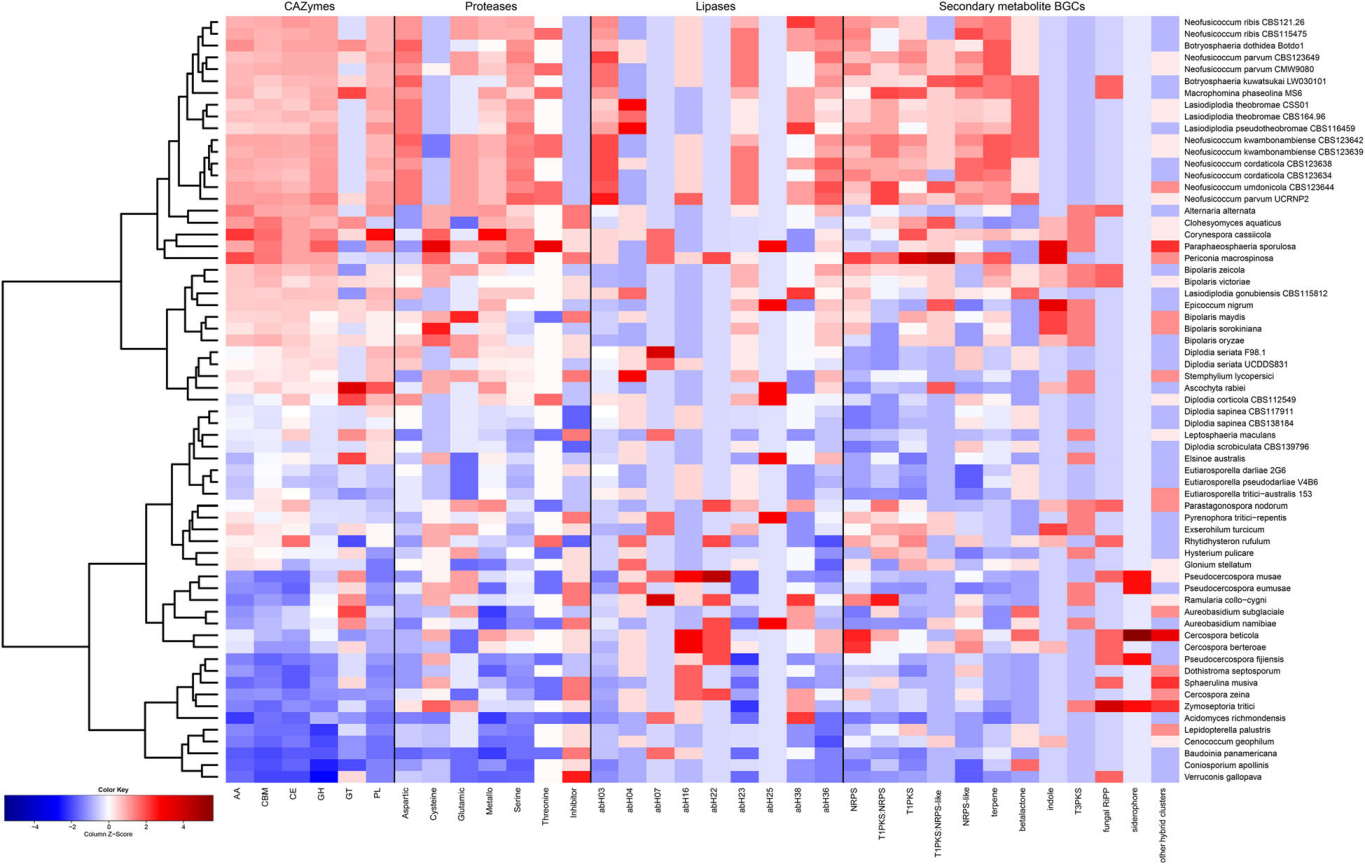
Hierarchical clustering and heatmap of *Botryosphaeriaceae* and other representative Dothideomycetes species based on the number of functional annotation categories of secreted and secondary metabolite BGCs. Overrepresented (red and dark red) and underrepresented (blue and dark blue) values are scaled relative to the column mean

Hierarchical clustering separated the taxa into four groups (Fig. 3). The taxa in the Pleosporomycetidae and Dothideomycetidae generally clustered separately, however, there was no overall clustering based on taxonomic placement. *Botryosphaeriaceae* species were present in three of the four dominant clusters.

A first cluster included *B. dothidea, B. kuwatsukai, M. phaseolina, L. theobromae, L. pseudotheobromae* and *Neofusicoccum* spp., as well as several Pleosporales (*Alternaria alternata, Clohesyomyces aquaticus, Corynespora cassiicola Paraphaeosphaeria sporulosa* and *Periconia macrospinosa*). A second cluster mostly contained taxa from the Pleosporales (*Aschochyta rabiei*, *Bipolaris* spp.*, Epicoccum nigrum* and *Stemphylium lycopersici*), but also contained *D. seriata, D. corticola* and *L. gonubiensis*. A third cluster contained taxa from both the Dothideomycetidae and Pleosporomycetidae, as well as *D. sapinea, D. scrobiculata* and *Eutiarosporella* spp. A fourth cluster was dominated by taxa from the Dothideomycetidae with the exception of *L. palustris, C. geophilum, C. apollinis* and *V. gallopava.*

**Fig. 4:**
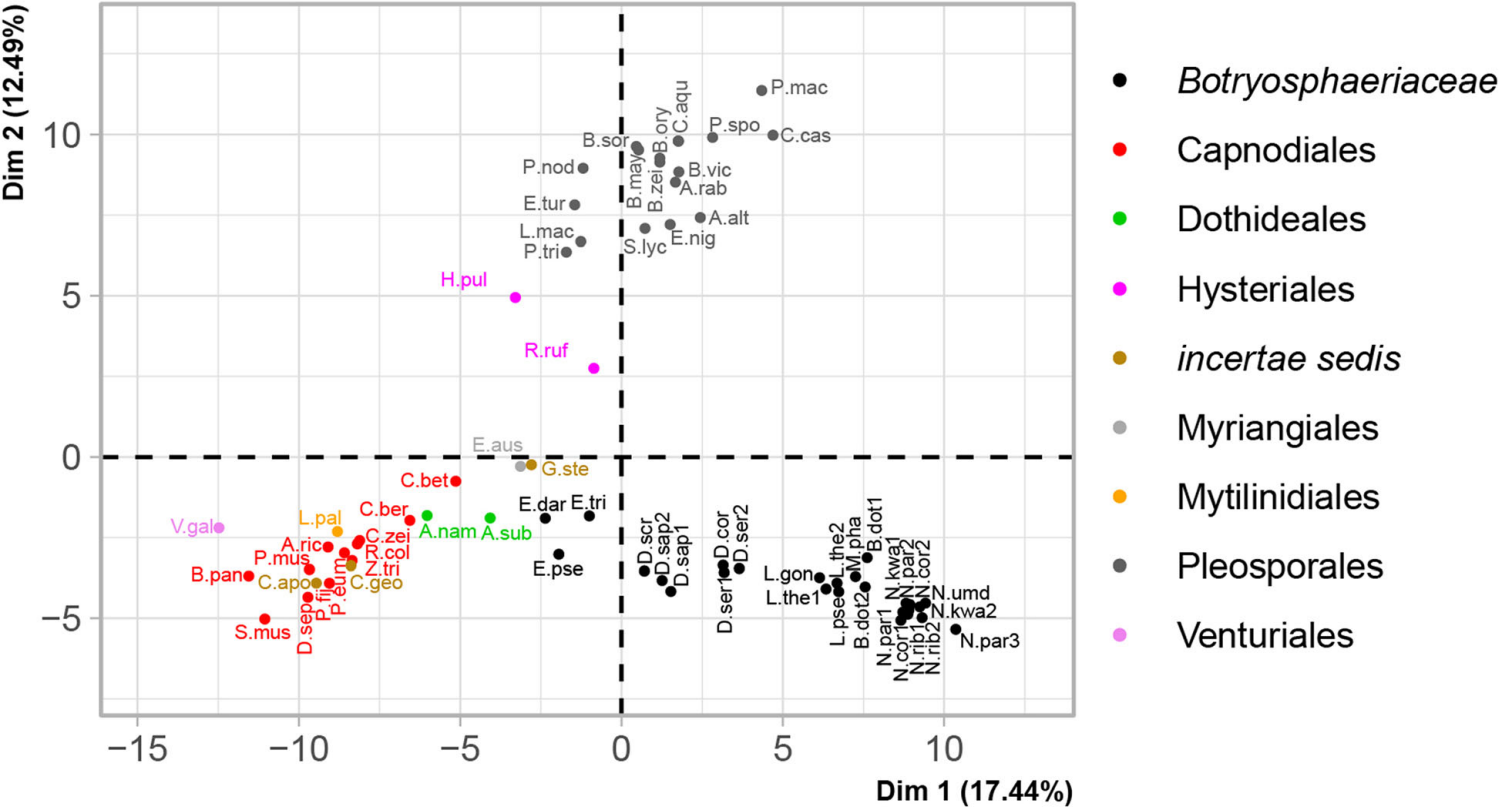
Principal component analysis of functional annotation categories of secreted proteins and secondary metabolite BGCs from *Botryosphaeriaceae* and other Dothideomycetes. Taxa are indicated using abbreviated names (Additional file 1) and colours indicate their Order/Family. The percentage variation accounted for by each principal component is indicated at each axis. The *Botrosphaeriaceae* varied mostly based on Dimension 1, with *Eutiarosporella* spp. clustered at the lower ranges of the first dimension (x-axis) followed by clusters accomodating the *Diplodia* species towards the middle ranges and the other genera of *Botryosphaeriaceae* clustered at the high ranges of the x-axis

PCA of the functional annotation categories clustered the data along 65 dimensions/principal components. The first two dimensions (Fig. 4) accounted for 29.9% of the variance among the taxa. The first dimension accounted for 17.4% and the second dimension for 12.5% of the variance. The first dimension was most strongly influenced by several secreted CAZyme (AA3, CBM1, GH131, PL3, CBM13, CBM18, CE8, CE12, AA7, GH43, PL4, PL1, CE5 and CBM63), cutinase (abH36) and lipase (abH03 and abH23) and protease (S09 and A01) families, as well as terpene BGCs. The second dimension was most strongly influenced by CAZyme (GH145, CBM3, GH6, GH11, PL26, CBM60, GH7, AA12, AA9, CBM2, GH16, CBM6, CBM87 and CE18), protease (S01, M14 and M36) families, as well as the indole and T3PKS BGC types.

The *Botryosphaeriaceae* were distributed mainly along the first dimension of the PCA and clustered into three groups. *Eutiarosporella* spp. clustered at the lower ranges of the first dimension (x-axis) followed by clusters accomodating the *Diplodia* species towards the middle ranges and the other genera of *Botryosphaeriaceae* clustered at the high ranges of the x-axis. The *Botryosphaeriaceae* clustered along a relatively narrow range along the second dimension (y-axis) compared to the other Dothideomycetes. The clustering of the other Dothideomycetes along the x-axis was correlated to their clustering on the y-axis: taxa towards the higher end of the x-axis also occurred towards the higher end of the y-axis. The clustering of taxa did not correspond to their nutritional lifestyle, but their taxonomic placement was reflected in their clustering.

### Genome architecture

**Fig. 5:**
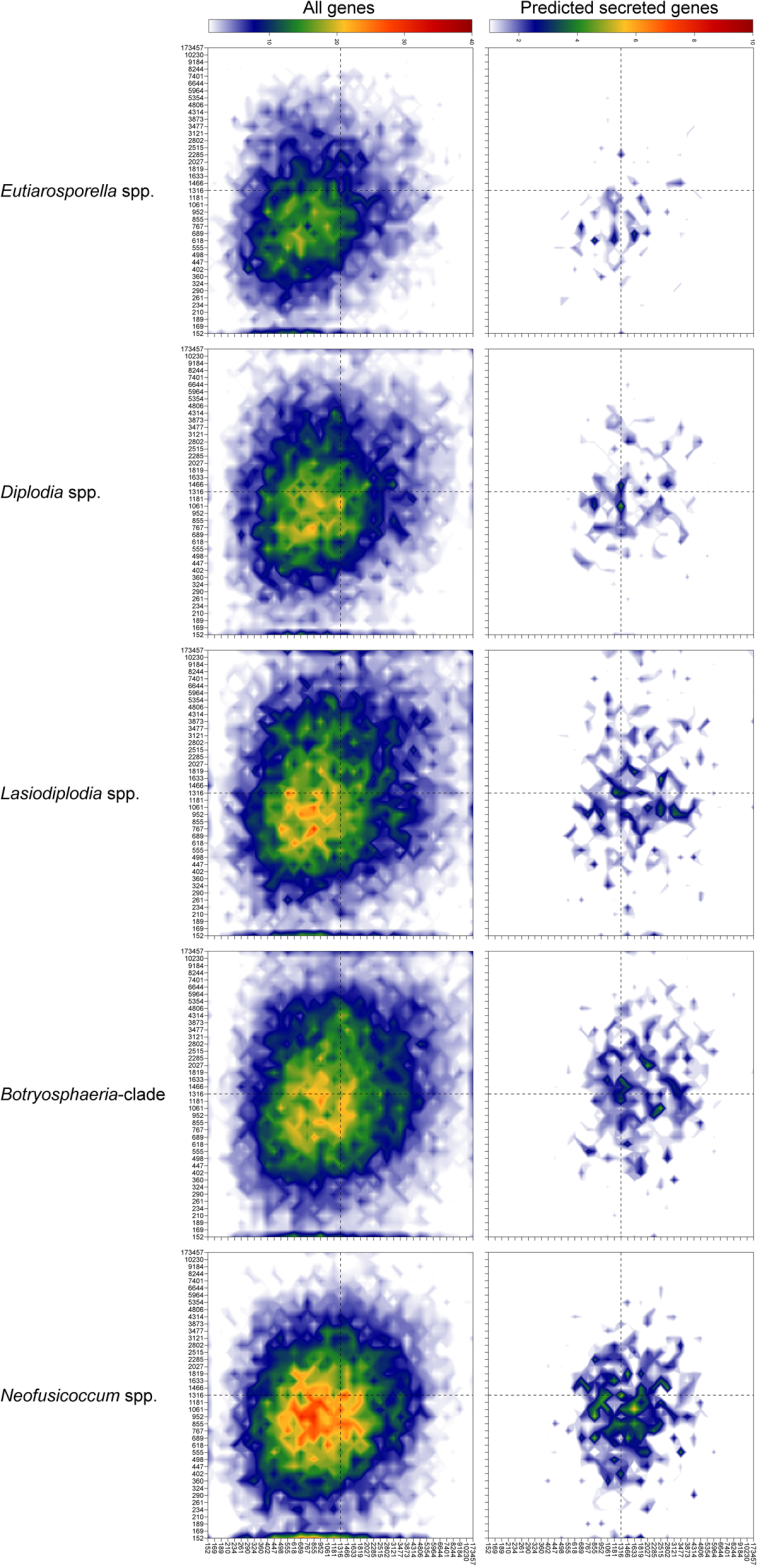
Gene density landscape of the genera of *Botryosphaeriaceae*. The two-dimensional heatmap shows the distribution of genes to gene dense or sparse regions of the genome based on their 5′ and 3′ flanking intergenic regions (FIRs). Two-dimensional heatmaps of each genus is the average across bins of all genomes in the group. The heatmaps labelled as “*Botryosphaeria-*clade” include the genomes of *B. dothidea, B. kuwatsukai* and *M. phaseolina*. The values on the axes are distances in base pairs and signify the upper limit of each bin. The median bin is indicated by dotted lines to assist in comparison between plots. The colours of the heatmap represents the number of genes present within each two-dimensional bin. All *Botryosphaeriaceae* had unimodal gene density distributions, with *Eutiarosporella, Diplodia* and *Neofusicoccum* with a greater overall gene density (smaller intergenic regions) than the other genera

Two-dimensional heatmaps of the 5′ and 3′ FIRs of the 26 *Botryosphaeriaceae* genomes indicated no genome compartmentalization (Fig. 5 and Additional file 4). This was evident from the unimodal gene density distributions of these genomes. Genomes of *Botryosphaeria, Lasiodiplodia* and *Macrophomina* had a higher proportion of genes in gene sparse regions than the other species in this family. *Eutiarosporella* spp., *D. sapinea*, *D. scrobiculata* and *Neofusicoccum* spp. had fewer genes in gene sparse regions.

The predicted secreted proteins of the *Botryosphaeriaceae* contained a greater number of genes in gene sparse regions than the total predicted genes (Additional file 4). The total CAZymes contained a higher proportion of genes in gene sparse regions than the total predicted genes. The secreted CAZyme gene density distribution was very similar to that of the total CAZymes. The secreted lipases and cutinases, however, occurred more frequently in gene sparse regions than the total lipases and cutinases (Additional file 4). This trend was also observed for the secreted proteases, although not as strongly. Genes associated with secondary metabolite BGCs were less prevalent in gene sparse regions.

The levels of repetitive sequences for most *Botryosphaeriaceae* genomes were between 3 and 8% of the total genome size (Table 5). The two *Botryosphaeria* spp. differed considerably in their repeat content (3.48 vs. 11.88%). The genome of *M. phaseolina* also had a higher than average repeat content (16.37%). Among the *Neofusicoccum* species, the genomes of *N. parvum* and *N. umdonicola* had less repetitive sequences than the other genomes of this genus. The genomes of *D. sapinea* and *D. scrobiculata* also contained less repetitive sequences than the other two *Diplodia* species.

**Table 5:** Summary of genomic architecture features of *Botryosphaeriaceae* genomes

	Genome size (Mb)	Repetitive sequences (bp)	% of repetitive sequences	Genome GC %	% genome that is GC rich (> 50%)	Genes in TA rich regions	Secreted genes in TA rich regions	% genome affected by RIP	Number of LRARs	Total size (bp) of all LRARs
*Eutiarosporella darliae* 2G6	27.27	1,415,283	5.19	61	98.3	3	0	0.91	4	32,276
*E. pseudodarliae* V4B6	26.74	1,290,421	4.83	61.53	98.6	7	0	0.4	0	0
*E. tritici-australis* 153	26.59	1,544,103	5.81	61.66	97.3	0	0	2.54	14	95,508
*Diplodia sapinea* CBS117911	36.05	1,344,948	3.73	56.84	95	73	4	1.12	2	9500
*D. sapinea* CBS138184	35.24	1,305,464	3.7	56.73	94.7	68	6	0.96	2	12,500
*D. scrobiculata* CBS139796	34.93	1,110,716	3.18	57.01	95.6	63	6	0.82	1	13,000
*D. seriata* UCDDS831	37.27	1,711,911	4.61	56.6	96.8	29	2	2.63	21	125,537
*D. seriata* F98.1	37.12	1,593,160	4.27	48.47	94.5	51	1	1.87	34	554,000
*D. corticola* CBS112549	34.99	2,102,085	6.01	47.92	94.5	31	3	3.39	42	690,014
*Lasiodiplodia theobromae* CBS164.96	42.97	1,498,597	3.49	54.74	89.9	354	32	0.72	3	59,500
*L. theobromae* CSS01	43.28	1,543,231	3.57	48.15	89.8	306	33	1.04	18	375,000
*L. pseudotheobromae* CBS116459	43.01	1,402,977	3.26	54.66	89.2	377	36	0.92	12	375,000
*L. gonubiensis* CBS115812	41.14	1,330,240	3.23	54.72	92.1	223	24	1.18	12	345,500
*Botryosphaeria dothidea* CMW8000	43.5	1,515,197	3.48	54.3	89.8	385	53	0.88	1	5000
*B. kuwatsukai* LW030101	47.39	5,628,118	11.88	53.09	81.1	406	41	9.65	170	1,387,657
*Macrophomina phaseolina* MS6	48.88	8,000,688	16.37	52.33	80.2	228	23	12.92	172	2,658,966
*Neofusicoccum cordaticola* CBS123634	45.71	3,594,350	7.86	54.9	86.5	566	54	3.7	105	1,071,347
*N. cordaticola* CBS123638	43.56	3,614,552	8.3	55.92	88.7	393	40	3.99	47	324,966
*N. kwambonambiense* CBS123639	44.17	3,369,216	7.62	55.92	89.1	408	40	2.75	64	693,395
*N. kwambonambiense* CBS123642	44.21	3,592,960	8.13	56.04	89.2	459	49	2.35	56	554,628
*N. parvum* CMW9080	41.41	1,944,617	4.7	56.54	91.3	406	40	1.36	11	63,557
*N. parvum* CBS123649	42.16	2,032,314	4.82	56.06	91.8	360	36	1.65	14	88,222
*N. parvum* UCRNP2	42.52	2,167,908	5.1	56.76	91.4	407	35	1.59	11	72,874
*N. ribis* CBS115475	43.18	3,241,261	7.51	55.71	89.6	379	38	4.52	79	911,957
*N. ribis* CBS121.26	43.12	3,171,232	7.35	55.88	89.1	423	51	4.71	25	153,638
*N. umdonicola* CBS123644	42.29	2,237,372	5.29	56.51	91.6	415	48	1.38	11	76,232

On average, the *Botryosphaeriaceae* genomes had less than 10% of their genomes composed of TA rich regions (GC < 50%) (Table 5). The genomes of *B. kuwatsukai* LW030101 and *M. phaseolina* had 18.9 and 19.8% of their genomes as TA rich regions, respectively. The genomes of *Diplodia* and *Eutiarosporella* had fewer TA rich regions (approximately 5% of the genome) compared to the other genera. Less than 5% of genes were present in TA rich regions and secreted genes were not found to be over-represented among these genes (Additional file 4). The genomes of *Diplodia* and *Eutiarosporella* had considerably fewer genes associated with TA rich regions, than the other taxa of this family.

Analysis of the prevalence of RIP in the genomes of *Botryosphaeriaceae* indicated that this has occurred to varying degrees in these genomes (Table 5). *Neofusicoccum* spp. had between 1.36 and 4.71% of their genome affected by RIP. The level of RIP was similar between different genomes of the same *Neofusicoccum* species. *Neofusicoccum parvum* and *N. umdonicola* had lower proportions of RIP affected sequences than the other species of the genus. There was a large (> 10-fold) difference in the level of RIP between *B. dothidea* and *B. kuwatsukai*. The genome of *M. phaseolina* had the highest (12.92%) amount of RIP of all the *Botryosphaeriaceae*. *Lasiodiplodia* spp. had RIP levels between 0.72 and 1.18%. The level of RIP in the genomes of *D. sapinea* and *D. scrobiculata* was lower than in *D. seriata* and *D. corticola*. The genome of *E. tritici-australis* had more than double the level of RIP affected regions than the other two species of the genus.

## Discussion

This study represents the first large-scale comparative genomics-level consideration of all available genomes of *Botryosphaeriaceae.* The results showed that the included *Botryosphaeriaceae* genomes, especially those of *Botryosphaeria*, *Macrophomina*, *Lasiodiplodia* and *Neofusicoccum*, encode high numbers of secreted hydrolytic enzymes and secondary metabolite BGCs. This emerges due to these fungi having increased numbers of genes associated with plant interactions in their secretome. The results also indicate that the *Botryosphaeriaceae* are most similar to species of the Pleosporomycetidae based on secreted enzyme and secondary metabolite profiles. *Botryosphaeriaceae* genomes were furthermore determined not to be compartmentalized based on gene density or GC-content.

There was a strong correlation between the number of hydrolytic enzymes and secondary metabolite BGCs, and the genome size and gene number of the *Botryosphaeriaceae* considered in this study. This correlation between genome size and gene number has generally not been seen in other fungi [4, 11, 80], because transposable elements and repetitive DNA vary significantly among species [11]. A recent comparison of Dothideomycetes genomes [81] also showed that genome size and gene number were not correlated to the abundance of functional annotation classes; neither to the lifestyle or phylogenetic placement of a species.

The genomes of *Botryosphaeria*, *Macrophomina*, *Lasiodiplodia* and *Neofusicoccum* had abundant secreted hydrolytic enzyme and secondary metabolite BGCs. This is a pattern that is most similar to prominent necrotrophic plant pathogens (*A. alternata, C. casiicola*), saprobes (*C. aquaticus*, *P. sporulosa*) and the endophyte/latent pathogen *P. macrospinosa* in the Pleosporales. The pattern was consistent with reports that necrotrophic pathogens tend to have higher numbers of hydrolytic enzymes and secondary metabolite toxins than biotrophs and symbiotic fungi [7, 82]. An abundance of secreted hydrolytic enzymes and secondary metabolite BGCs found in the *Botryosphaeriaceae* is also similar to that of other species of woody endophytes. Studies on such endophytic species have shown that they have similar or higher amounts of various secreted enzymes (notably plant cell wall degrading enzymes) or secondary metabolites than closely related plant pathogenic species [41–44]. The specific gene families that are enriched, however, differ among endophytic lineages, due to the evolutionary independent origins of endophytism [41–44]. It has furthermore been noted that fungi with dual lifestyles (e.g. fungi with endophytic and pathogenic phase) have large numbers of CAZymes [83, 84], however, few studies have investigated this. These observations also emerging from the present study are consistent with the known dual-lifestyle of *Botryosphaeriaceae* as latent pathogens.

The *Botryosphaeriaceae* genomes were rich in CAZymes, especially those involved in plant cell wall degradation (PCWD), although at lower numbers in the genomes of *Diplodia* and *Eutiarosporella* species. CAZymes involved in the degradation of cellulose, hemicellulose and pectin were present in all *Botryosphaeriaceae.* The genomes of *Botryosphaeria*, *Macrophomina*, *Lasiodiplodia* and *Neofusicoccum* were particularly rich in CAZyme families involved in cell wall degradation. Specifically, CAZymes involved in plant, general and fungal cell wall degradation [4, 85, 86] were abundant in the genomes of the above-mentioned genera.

The *Botryosphaeriaceae* secretomes were rich in CAZyme families involved in the recognition of cellulose (CBM1) and chitin (CBM18). Although carbohydrate-binding domains have no catalytic activity of their own they play important roles in substrate recognition and binding of other CAZymes [87, 88], they are also involved in the protection of fungal cell walls from degradation by host enzymes and prevention of host detection [1, 89]. High numbers of secreted CAZymes involved with PCWD have also been found in previous studies of *N. parvum* and *D. seriata* [31, 32] and in other, especially necrotrophic, Dothideomycetes [62, 63, 66, 70, 85]. The abundance of these CAZyme families in some genera of *Botryosphaeriaceae* suggests that cell wall degradation plays an important role in the biology of these fungi.

Several important CAZyme families that are common among Dothideomycetes were absent from all the *Botryosphaeriaceae* genomes, i.e. Acetyl xylan esterase (CE3) [90], Pyrroloquinoline quinone-dependent oxidoreductase (AA12) [91], endo-α-1,4-polygalactosaminidase (GH114) [92] and α-L-arabinofuranosidase/β-xylosidase (GH54) [93]. The absence of these CAZyme families in the *Botryosphaeriaceae* does not necessarily indicate a gap in the metabolic repertoire of these fungi because a large degree of functional redundancy is commonly seen in fungal CAZyme repertoires [94–96]. Interestingly, some of the CAZyme families that can functionally compensate for the absence of the above-mentioned CAZyme families are those that were found to be among the most abundant secreted CAZyme families of the *Botryosphaeriaceae* (e.g. CE16, AA3, AA7, GH15 and GH3).

The *Botryosphaeriaceae* were rich in secreted serine-, metallo- and aspartic-proteases. Protease families (A01, S08, S09, S10) that were previously identified as the most common secreted proteases among Dothideomycetes [4] were also the most abundant in the *Botryosphaeriaceae*. Secreted proteases play important roles in nutrient acquisition, signalling and degradation of plant defences [3, 97–99]. Although secreted proteases are abundant in several necrotrophic pathogens, e.g. *Corynespora cassiicola* [66] and several *Colletotrichum* spp. [100], no patterns between nutritional lifestyle and the abundance of secreted proteases could be distinguished. The precise function of most of these proteases in the *Botryosphaeriaceae* are unknown and their role during infection and disease expression remains to be determined.

A lower abundance and diversity of secreted protease inhibitors of the *Botryosphaeriaceae* suggests a reduced capacity and/or need for extracellular enzyme inhibition. Plant pathogenic fungi secrete protease inhibitors to inhibit plant proteases involved in defence responses [101] and several protease inhibitors are known virulence factors, e.g. *avr2* of *Cladosporium fulvum* [102, 103] and *Pit2* of *Ustilago maydis* [104]. However, the exact role of many fungal protease inhibitors, such as those secreted by the *Botryosphaeriaceae* and Dothideomycetes, remains unknown [105].

*Botryosphaeriaceae*, especially species of *Botryosphaeria*, *Macrophomina*, *Lasiodiplodia* and *Neofusicoccum* possessed high numbers of secreted lipases. Three lipase families were present in high numbers in the secretomes of the Dothideomycetes, i.e. *Candida rugosa* lipase-like (abH03), cutinases (abH36 and CE5) and Filamentous fungi lipases (abH23). The *Botryosphaericeae* genomes were rich in secreted enzymes for these three families. Lipases and cutinases are important for fungal penetration of host tissue [6, 106], growth and adhesion [107, 108] and manipulation of host defences [5]. The abundance of these secreted lipases and cutinases emphasises their potentially important role during the infection process in the *Botryosphaeriaceae*.

The genomes of *Botryosphaeria*, *Macrophomina*, *Lasiodiplodia* and *Neofusicoccum* contained many BGCs, especially t1PKS, NRPS, NRPS-like and TS type clusters. The products produced by most of these clusters are unknown, however, the products of some clusters could be determined. These compounds included melanin, phytotoxins (ACT-Toxin II, (−)-Mellein), siderophores (dimethylcoprogen) and antioxidants (pyranonigrin E). Many phytotoxic secondary metabolites have been identified in the *Botryosphaeriaceae* [109–114]. Of these, the most commonly identified phytotoxic compounds are mellein and its derivatives [115, 116]. The presence of a predicted ACT-toxin producing gene cluster in the *Botryosphaeriaceae* is interesting as it is a host selective toxin from citrus infecting *A. alternata* [117]. This toxin is part of the Epoxy-decatrienoic acid (EDA) family, however, neither this type of toxin or any in this family has been isolated from the *Botryosphaeriaceae*. The ability to produce secondary metabolite toxins have been associated with pathogenic fungi’s lifestyle, host range and virulence [4, 118, 119]. Many plant pathogenic fungi have large numbers of secondary metabolite BGCs, e.g. *Bipolaris* spp. [120], *Corynespora cassiicola* [66], *Colletotrichum* spp. [100] and *Pyrenophora teres* [4], but so also do fungi with other lifestyles such as the saprobic *Annulohypoxylon stygium* [121], *Hysterium pulicare,* and *Rhytidhysteron rufulum* [4]. Despite the observation that the total abundance of secondary metabolite BGCs does not predict lifestyle, several fungal toxins are able to modulate a fungal species’ host range or virulence, e.g. the AF-toxin of *A. alternata* [118]*,* the *Hybrid-1,2* and *3* genes of *Eutiarosporella darliae* and *E. pseudodarliae* [27] and the HC-toxins of *Bipolaris zeicola* [122].

The *Botryosphaeriaceae* genera were shown to possess non-compartmentalized genomes. These species were characterized as having moderate to high % GC, RIP-affected genomes with low amounts of repetitive DNA, a slight preferential localization of secreted genes to gene sparse regions and no preferential localization of secreted genes to TA rich regions. Most Dothideomycetes do not have compartmentalized genomes, but several important pathogenic species (e.g. *L. maculans* and *Pseudocercospora* spp.), have genomes with high levels of repetitive DNA (often transposable elements) [12, 57] enriched for fast-evolving genes related to pathogenicity or virulence [57, 123, 124]. However, not all rapidly evolving fungal phytopathogens have this characteristic ‘two-speed’ genome architecture [13]. Where ‘two-speed’ genomes rely on the action of leaky RIP to generate variation for selection to act on, ‘one-speed’ genomes of fast-evolving plant pathogens rely on the absence of RIP that allows gene duplication/copy number variation to generate variation [13]. The *Botryosphaeriaceae* are not like those species with ‘two-speed’ genomes as they don’t have compartmentalized genomes, but RIP is also not completely absent as seen in species with ‘one-speed’ fast-evolving genomes.

## Conclusions

This study is the first large-scale comparative genomics study to consider all available genomes of *Botryosphaeriaceae*. It has illustrated large variability in the secreted hydrolytic enzyme and secondary metabolite biosynthetic repertoire between genera of this family. Most importantly, we have demonstrated similarities between the *Botryosphaeriaceae* and necrotrophic plant pathogens and endophytes of woody plants, emphasising their role as latent pathogens. This study highlights the importance of these genes in the infection biology of *Botryosphaeriaceae* species and their interaction with plant hosts. This knowledge will be useful in future studies aimed at understanding the mechanisms of endophytic infections and how these transition to a pathogenic state. The results should also help to better understand the genetic factors involved in determining the complex question of host range in the *Botryosphaeriaceae*.

## Materials and methods

### Genomic data

All available, published *Botryosphaeriaceae* genomes were retrieved from public databases (NCBI and JGI). Additionally, we sequenced and assembled 12 genomes representing three *Lasiodiplodia* spp. and five *Neofusicoccum* spp. (Table 1). To standardize protein annotations for downstream application, all of the above *Botryosphaeriaceae* genomes were annotated using the same pipeline described below. Additionally, the genomes and protein annotations of 41 Dothideomycetes and *Aspergillus nidulans* (Eurotiales), which had both genomic sequences and annotated protein sequences available on NCBI, were retrieved (Table 1). These genomes were used for comparative purposes in the phylogenomic analyses, hydrolytic enzyme and secondary metabolite BGC analyses and in the statistical clustering analyses, described below.

#### DNA extraction, genome sequencing and assembly

Cultures of three *Lasiodiplodia* and five *Neofusicoccum* species (Table 1) were inoculated onto cellophane covered 2% malt extract agar (MEA; Biolab, Merck) and incubated at 22 °C. After 5 days, mycelium was harvested from the surface of the cellophane using a sterile scalpel and DNA was extracted using a modified phenol/chloroform protocol, that included the addition of potassium acetate to precipate protein. Mycelium was ground to a fine powder using liquid nitrogen and mortar and pestle. Approximately 500 mg of ground mycelium was used for DNA extraction. To the ground mycelia, 18 ml of a 200 mM Tris-HCl (pH 8.0), 150 mM NaCl, 25 mM EDTA (Ethylenediaminetetraacetic acid, pH 8.0) and 0.5% SDS (Sodium dodecyl sulfate) solution and 125 μl of 20 mg/ml Proteinase K was added and incubated at 60 °C for 2 h. This was followed by addition of 6 ml of 5 M potassium acetate and 30 min incubation at 0 °C. Samples were then centrifuged at 5000 g for 20 min. The aqueous phase was kept and 24 ml of a 1:1 phenol:chloroform solution was added; samples were then centrifuged as above. Two chloroform washes were performed on the aqueous phase, followed by addition of 100 μl of 10 mg/ml Rnase A and incubated for 2 h. DNA was precipitated using one volume isopropanol and centrifuged for 30 min. The pellet was cleaned with two 70% ethanol washes and resuspended in 1 x Tris-EDTA buffer.

The extracted DNA was used for paired-end sequencing (average fragment size of 500 bp). All samples were sequenced on an Illumina HiSeq 2500 platform, except for *N. parvum* (isolate CMW9080) that was sequenced on a Miseq platform. The quality of the resulting reads was assessed using FastQC 0.10.1 [125] and low quality and short reads were trimmed or discarded using Trimmomatic 0.30 [126]. De novo genome assembly was performed by Velvet 1.2.10 [127] and Velvetoptimiser 2.2.5 [128]. Paired-end reads were used to scaffold the assembly and insert size statistics were determined by Velvet for each genome assembly. Genome assembly summary statistics were calculated with the AssemblyStatsWrapper tool of BBtools 38.00 [129].

#### Genome annotation

The twelve sequenced genomes described above, as well as the fourteen *Botryosphaeriaceae* genomes retrieved from public databases, were annotated as follows: Custom repeat libraries were constructed for each genome assembly using RepeatModeler 1.0.10 [130]. BRAKER 1.10 [131] was used to create trained GeneMark-ET 4.29 [132] and AUGUSTUS 3.2.3 [133] profiles using previously published *N. parvum* transcriptome data (SRR3992643 and SRR3992649) [31]. Genomes were annotated through the MAKER2 2.31.8 [134] pipeline using the custom repeat libraries and the BRAKER trained GeneMark-ET 4.29 and AUGUSTUS profiles. Genomes and genome annotations were assessed for completeness with BUSCO 4.0.5 [135] using the Ascomycota ortholog library (Creation date 2020-09-10, 1706 core orthologous genes). The annotations for six *Botryosphaeriaceae* genomes available on public databases prior to this study were also assessed using BUSCO and compared to those of the annotations generated in this study.

### Phylogenomic analyses

To illustrate the relationships between species and genera of the *Botryosphaeriaceae*, as well as the relationship of this family to the rest of the Dothideomycetes, a robust phylogeny was created from the genome data. Single copy core orthologous genes were identified from individual genomes (Table 1) using BUSCO (as described above). The BUSCO genes present in all taxa (207 genes) were selected for further analysis. Each orthogroup was aligned using MAFFT 7.407 [136] before being concatenated into a single matrix. RAxML 8.2.4 [137] was used to perform maximum likelihood phylogenetic inference using the PROTGAMMAAUTO option and a thousand bootstrap replicates were performed. Trees were rooted using sequences from *Aspergillus nidulans*.

### Functional annotation

The genome annotation data for the *Botryosphaeriaceae*, the other Dothideomycetes and the outgroup *A. nidulans* were used to perform functional annotation for hydrolytic enzymes and secondary metabolite BGCs. CAZymes were predicted by searching the total predicted proteins of each genome against the CAZy database [138] using dbCAN2 (HMMdb release v9.0) [139]. Only those CAZyme predictions supported by two or more tools (HMMR, DIAMOND, Hotpep) were retained. Proteases and protease inhibitors were predicted by subjecting the predicted protein sequences to a BLASTP [140] search against the MEROPS protease database 12.0 [141] using a cut off E-value of 1E-04. Lipases and cutinases were predicted by searching protein sequences against lipase and cutinase hidden Markov model (HMM) profiles retrieved from the Lipase Engineering Database v 3.0 [142] using HMMER 3.1b2 [143]. Secondary metabolite BGCs were identified from all annotated genomes using AntiSMASH v5.1.1 [144].

The total predicted proteins were also analyzed for the presence of signal peptides, involved in protein secretion. Phobius 1.01 [145] and SignalP 4.1 [146] were used to assess the presence of signal peptides and TMHMM 2.0 [147] was used to determine if any transmembrane regions occurred within these proteins. Only proteins with a signal peptide predicted by both Phobius and SignalP, as well as no transmembrane domains outside of the signal peptide predicted by both Phobius and TMHMM were regarded here as predicted secreted proteins.

Statistical analyses were performed using the total number of annotations, as well as the number of each enzyme class/secondary metabolite BGC type. Statistical tests were done comparing the genera of the *Botrysphaeriaceae,* but also for comparisons between Dothideomycetidae, Pleosporomycetidae and *Botryosphaeriaceae.* Due to the small number of representative genomes for *Botryosphaeria* (2) and *Macrophomina* (1) and their close phylogenetic placement, they were combined in a single group (i.e. *Botryosphaeria*-clade) for the purpose of statistical analyses. We further also included *Cenococcum geophilum, Coniosporium apollinis* and *Glonium stellatum* in the Pleosporomycetidae, based on their phylogenetic placement (Fig. 1).

We calculated pairwise Pearsons correlation coefficients [148] in order to test whether genome size, the number of predicted genes or any of the functional annotation categories were correlated. The Shapiro-Wilk test [149] was performed in order to test for a normal distribution using the byf.shapiro function of the RVAideMemoire R-package [150]. Pairwise comparisons to test for significant differences were done using one-tailed Wilcoxon rank-sum tests [151] in R using the pairwise.wilcox.test function of the R stats package [152] with either the ‘greater’ or ‘less’ alternative hypothesis options. This is a non-parametric test to determine whether two independent sets of values have the same distribution. Unlike the t-test, the Wilcoxon rank sum test does not assume a normal distribution [153] and thus was better suited to our data. These same tests as above were done using the total number of predicted, as well as the number of secreted genes, associated with hydrolytic enzyme families to test if specific CAZyme, protease or lipase families within the secretomes of the Dothideomycetidae, Pleosporomycetidae and *Botryosphaeriaceae* were significantly different.

### Analysis of gene family evolution

CAFE (Computational Analysis of gene Family Evolution) v4.2 [154] was used to study the evolution of gene family size in the hydrolytic enzymes and secondary metabolite BGCs. To this end, the phylogeny described above was converted to an ultrametric tree using r8s v1.81 [155]. This was then calibrated by fixing the age of the *Botrosphaeriaceae* to 61 million years [79] and constraining the age of the Dothideomycetes to 303–357 million years [156]. Gene family sizes for the total predicted CAZymes, proteases, lipases and secondary metabolite BGCs (Additional file 1) were used as input for the CAFE analyses. CAFE was run using separate lambda (birth) and mu (death) rate parameters. Additionally, two separate rate classes were allowed in that the rate parameters were calculated independently for the *Botryosphaeriaceae* and the remaining Dothideomycete taxa. CAFE was run using a *P*-value cutoff of 0.01 and Viterbi *P*-values were calculated to significant expansions/contractions across branches.

### Hierarchical clustering and principal component analysis

Hierarchical clustering was done to determine the similarity between taxa based on the secreted hydrolytic enzyme classes and secondary metabolite BGC types. The heatmap.2 function of the gplots [157] R package was used to perform the analysis. Additionally, principal component analysis (PCA) was performed using the same functional annotations as used in the hierarchical clustering, with the exception that for CAZymes and proteases the number of genes associated with each family (e.g AA1, A01) was used instead of those of each class (e.g AA, Aspartic). The FactoMineR [158] R package was used to perform the analysis and Factoshiny [159] was used to generate PCA plots.

### Genome architecture

Gene densities were analyzed for each *Botryosphaeriaceae* genome to assess the level of genome compartmentalization. Intergenic distances were used as a measure of gene density, by considering the 5′ and 3′ flanking intergenic regions (FIRs) of each gene. FIR lengths were used for two-dimensional data binning to construct gene density heat maps [160] using R [152]. Possible differences in the gene density distributions between the total and secreted CAZymes, proteases and lipases were also considered. We compared the relative amounts (i.e. the percentage) of these genes located in gene-sparse regions. In this case, gene sparse regions were defined as genes with both 5′ and 3′ FIRs larger than 1500 bp, as previously used by S Raffaele and S Kamoun [11].

The repeat contents of the *Botryosphaeriaceae* genomes were determined using RepeatMasker [161] with custom repeat libraries created by RepeatModeler. The presence of TA rich regions and the genes present therein were determined using OcculterCut [124]. Specifically, the numbers of the total secreted genes, the secreted CAZymes, proteases and lipases, as well as the number of secondary metabolite BGCs associated with TA rich regions were determined. The occurrence of RIP, including large regions affected by RIP (LRARs), was determined in each genome using TheRIPper [162].

## Supplementary Information

**Additional file 1.**

**Additional file 2.**

**Additional file 3.**

**Additional file 4.**

## Data Availability

All data generated or analysed during this study are included in this published article (and its supplementary information files). Genomic data used in this study (Table 1) are available from NCBI (https://www.ncbi.nlm.nih.gov/genome) and the JGI Genome Portal (http://genome.jgi.doe.gov). Raw sequencing reads of the de novo genomes described in this study linked to Bioproject PRJNA497969 (Biosample Accessions SAMN10319569 - SAMN10319580) have been deposited to the Sequence Read Archive (SRA, https://www.ncbi.nlm.nih.gov/sra) database and are awaiting accession numbers.
