# Supplementary figures and images for: Increased abundance of secreted hydrolytic enzymes and secondary metabolite gene clusters define the genomes of latent plant pathogens in the Botryosphaeriaceae
Source: BMC Genomics. 2021 Aug 4;22:589. doi: 10.1186/s12864-021-07902-w (PMC8336260; doi:10.1186/s12864-021-07902-w)

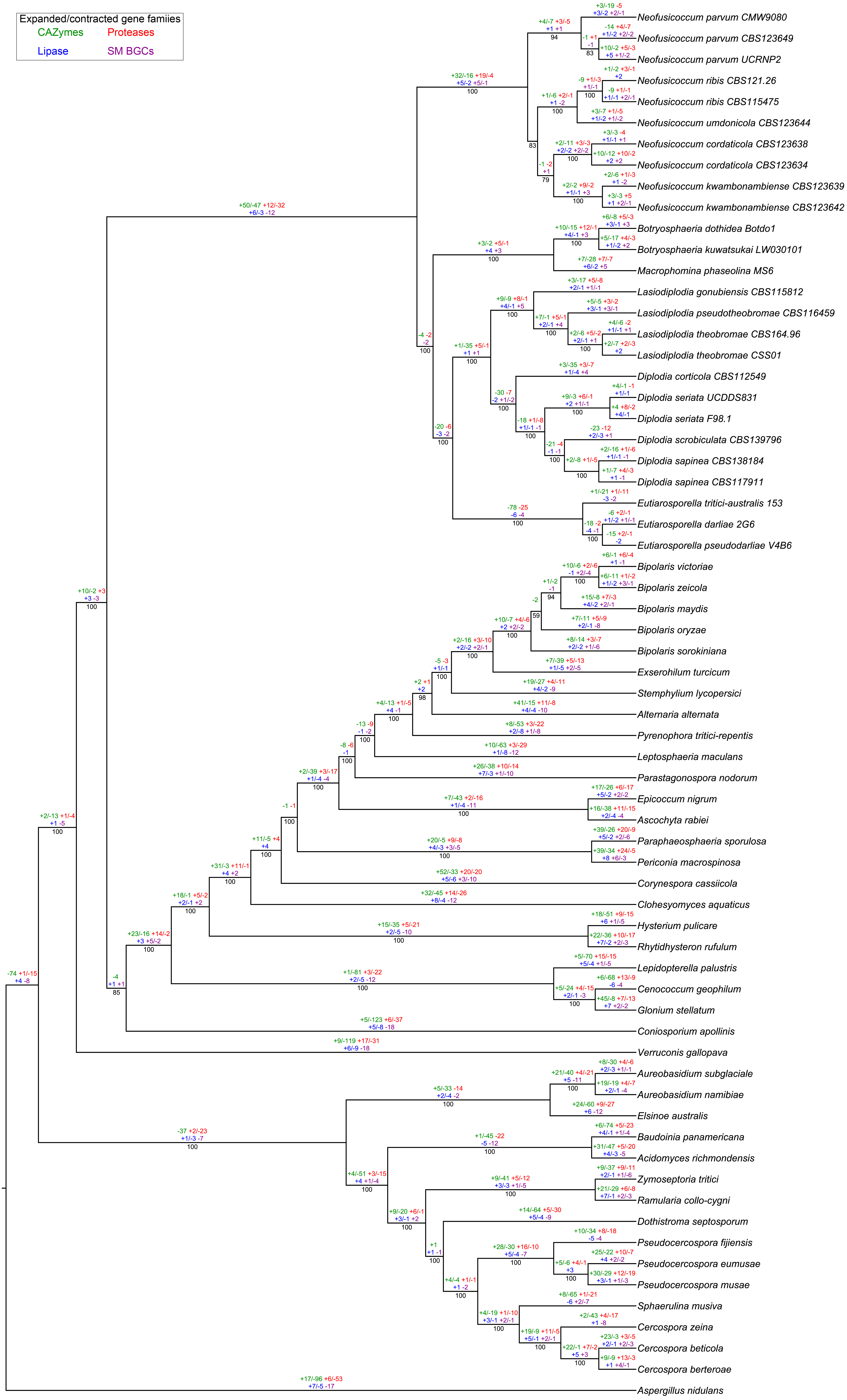

Supplement: Supplementary file 3 — Additional file 3. [file 12864_2021_7902_MOESM3_ESM.jpg]
